# Supplementary material for: Learning to select computations in recurrent neural circuits
Source: bioRxiv. 2026 Apr 16:2026.04.14.718499. Preprint. [Version 1] doi: 10.64898/2026.04.14.718499 (PMC13104915; doi:10.64898/2026.04.14.718499)
Supplement: Supplement 1 [file NIHPP2026.04.14.718499v1-supplement-1.pdf]

bioRxiv preprint doi: https://doi.org/10.64898/2026.04.14.718499; this version posted April 16, 2026. The copyright holder for this preprint (which was not certified by peer review) is the author/funder, who has granted bioRxiv a license to display the preprint in perpetuity. It is made available under aCC-BY 4.0 International license.

6 Supplementary Information

6.1 Supplementary figures for the simple choice task in Callaway et al. (2021)

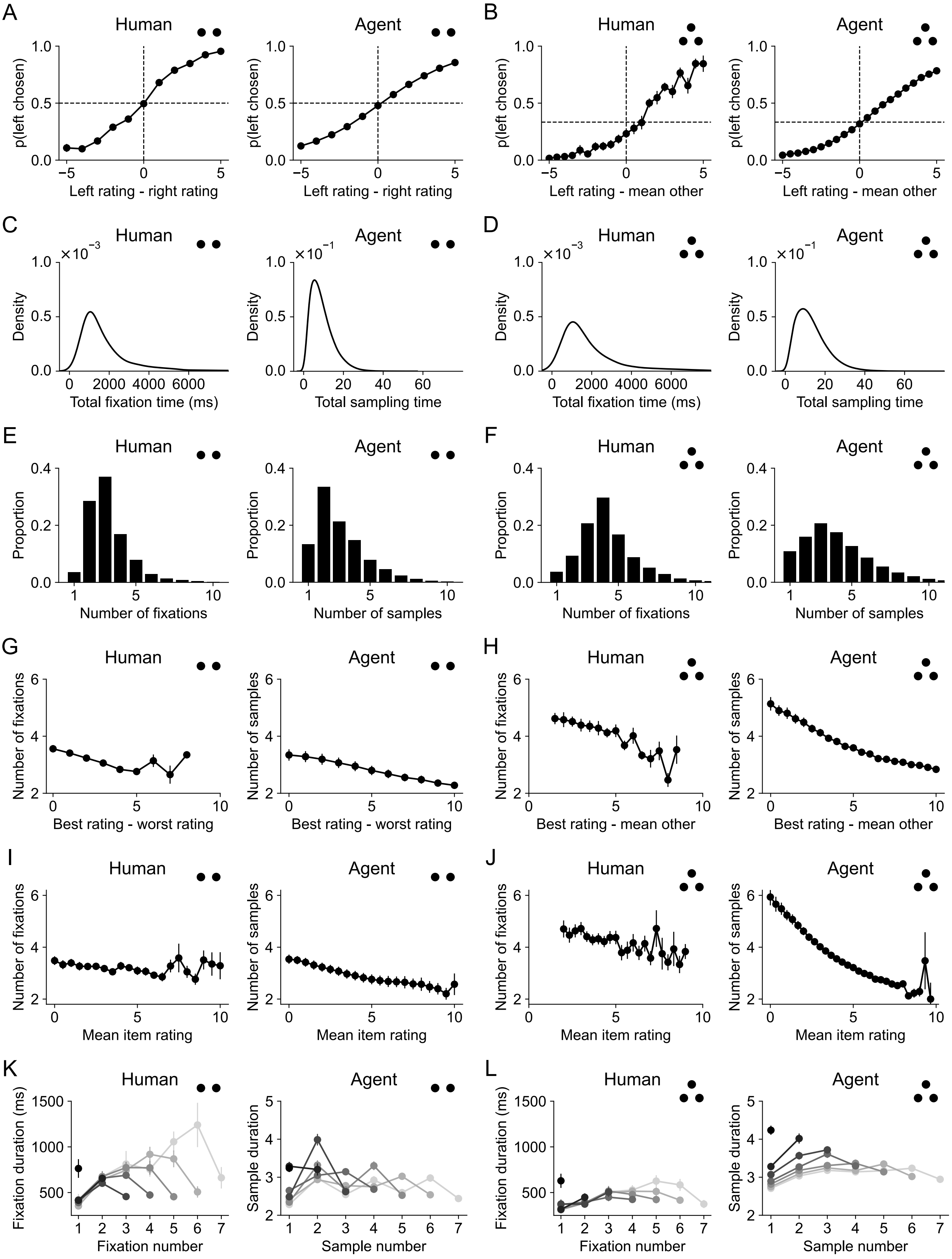

bioRxiv preprint doi: https://doi.org/10.64898/2026.04.14.718499; this version posted April 16, 2026. The copyright holder for this preprint (which was not certified by peer review) is the author/funder, who has granted bioRxiv a license to display the preprint in perpetuity. It is made available under aCC-BY 4.0 International license.

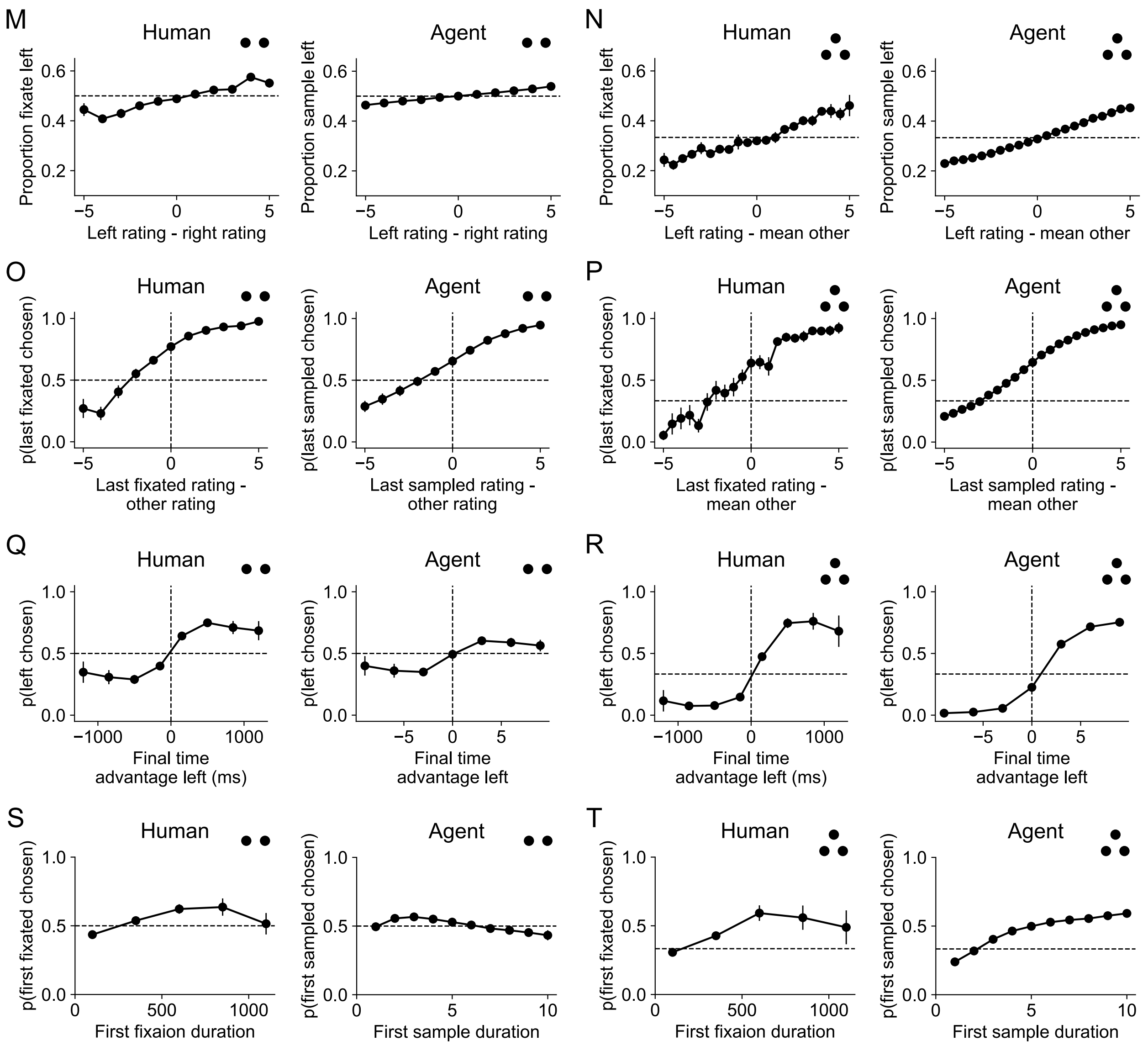

Figure S1: **Behaviors from humans and the meta-RL agent on the simple choice task.** (A–B) Choice probability as a function of relative rating. (C–D) Distribution of total fixation or sampling time. (E–F) Histogram of number of fixations or samples in a trial. Here a sample refers to a contiguous period of sampling from the same item, analogous to a fixation in humans. (G–H) Number of fixations or samples as a function of relative rating. (I–J) Number of fixations or samples as a function of mean rating. (K–L) Fixation or sample duration as a function of fixation or sample number. Colors correspond to number of total fixations or samples within a trial. (M–N) Proportion of time attending to the left item as a function of its relative rating. (O–P) Probability that the last attended item is chosen as a function of its relative rating. (Q–R) Probability that the left item is chosen as a function of its final time advantage, given by total fixation or sampling time to the left item minus the mean total fixation or sampling time to the other item(s). (S–T) Probability of choosing the first fixated item as a function of first fixation or sample duration.

bioRxiv preprint doi: https://doi.org/10.64898/2026.04.14.718499; this version posted April 16, 2026. The copyright holder for this preprint (which was not certified by peer review) is the author/funder, who has granted bioRxiv a license to display the preprint in perpetuity. It is made available under aCC-BY 4.0 International license.

6.2 Supplementary figures for the simple choice task in Callaway et al. (2021)

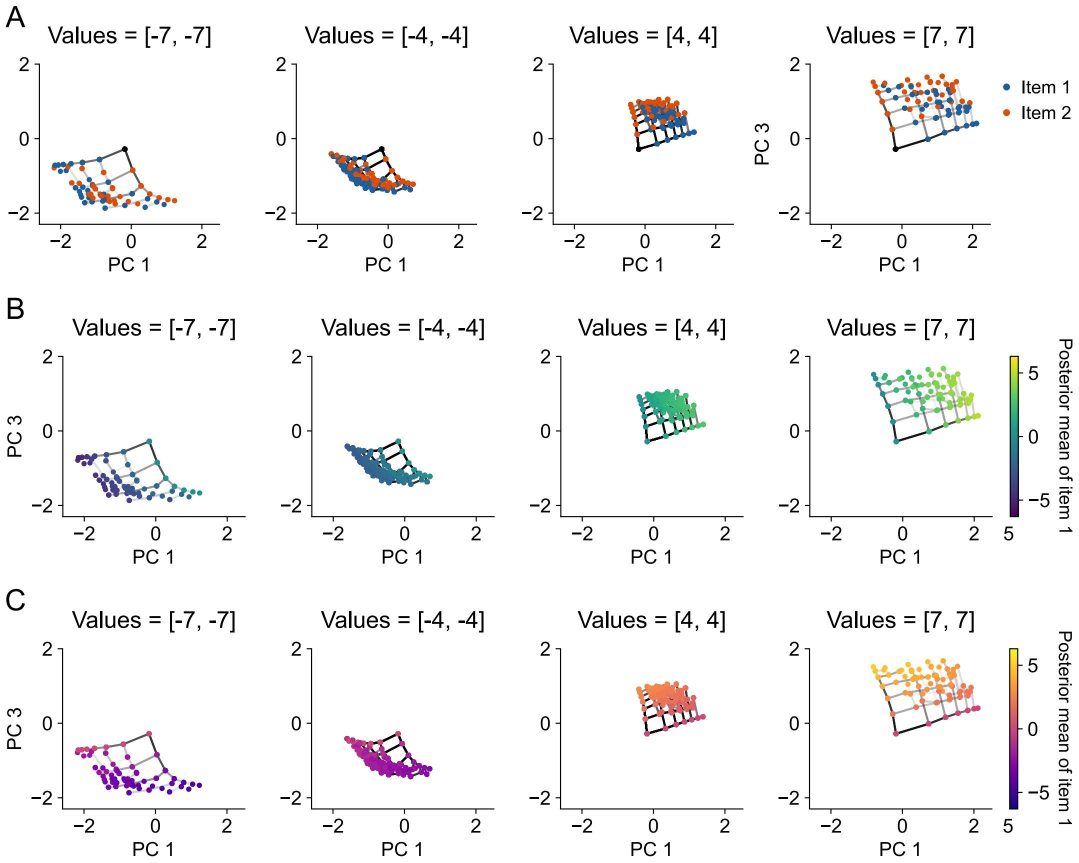

Figure S2: **PCA results for different value conditions in the binary choice task**. Hidden states are colored by the current attended item (A), posterior mean of item 1 (B), and posterior mean of item 2 (C). Panels show conditions where both items have values of -7, -4, 4, and 7, respectively. Line transparency shows transition frequency. Hidden states are simulated with noise-free value samples for visualization (Methods).

bioRxiv preprint doi: https://doi.org/10.64898/2026.04.14.718499; this version posted April 16, 2026. The copyright holder for this preprint (which was not certified by peer review) is the author/funder, who has granted bioRxiv a license to display the preprint in perpetuity. It is made available under aCC-BY 4.0 International license.

6.3 Supplementary figures for the planning task in Callaway et al. (2024b)

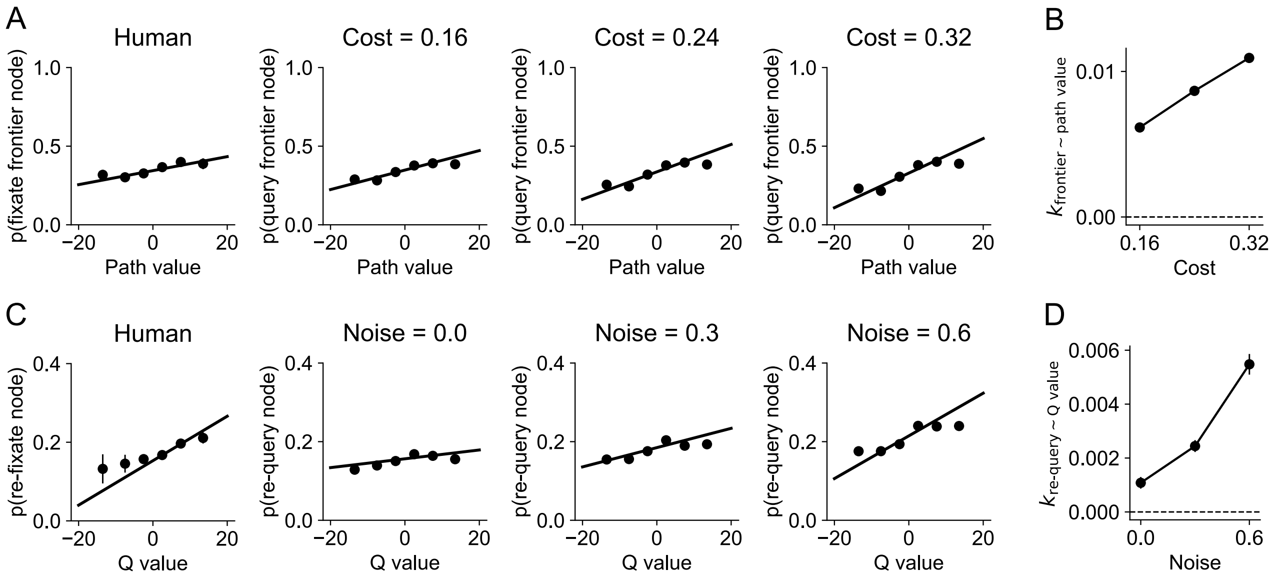

Figure S3: **Planning strategy under different levels of search cost and memory capacity.** (A) Probability of attending to a frontier state as a function of it path value for humans and agents with different costs. (C) Correlation coefficient between frontier fixation/query and path value as a function of cost per query step. (E) Probability of re-attending to a state as a function of its Q value for humans and agents with different noise levels in hidden states. (F) Correlation coefficient between re-fixation/re-query and Q value as a function of noise. Here noise refers to the parameter α that specifies the proportion of hidden state variance replaced by noise (Methods).

bioRxiv preprint doi: https://doi.org/10.64898/2026.04.14.718499; this version posted April 16, 2026. The copyright holder for this preprint (which was not certified by peer review) is the author/funder, who has granted bioRxiv a license to display the preprint in perpetuity. It is made available under aCC-BY 4.0 International license.

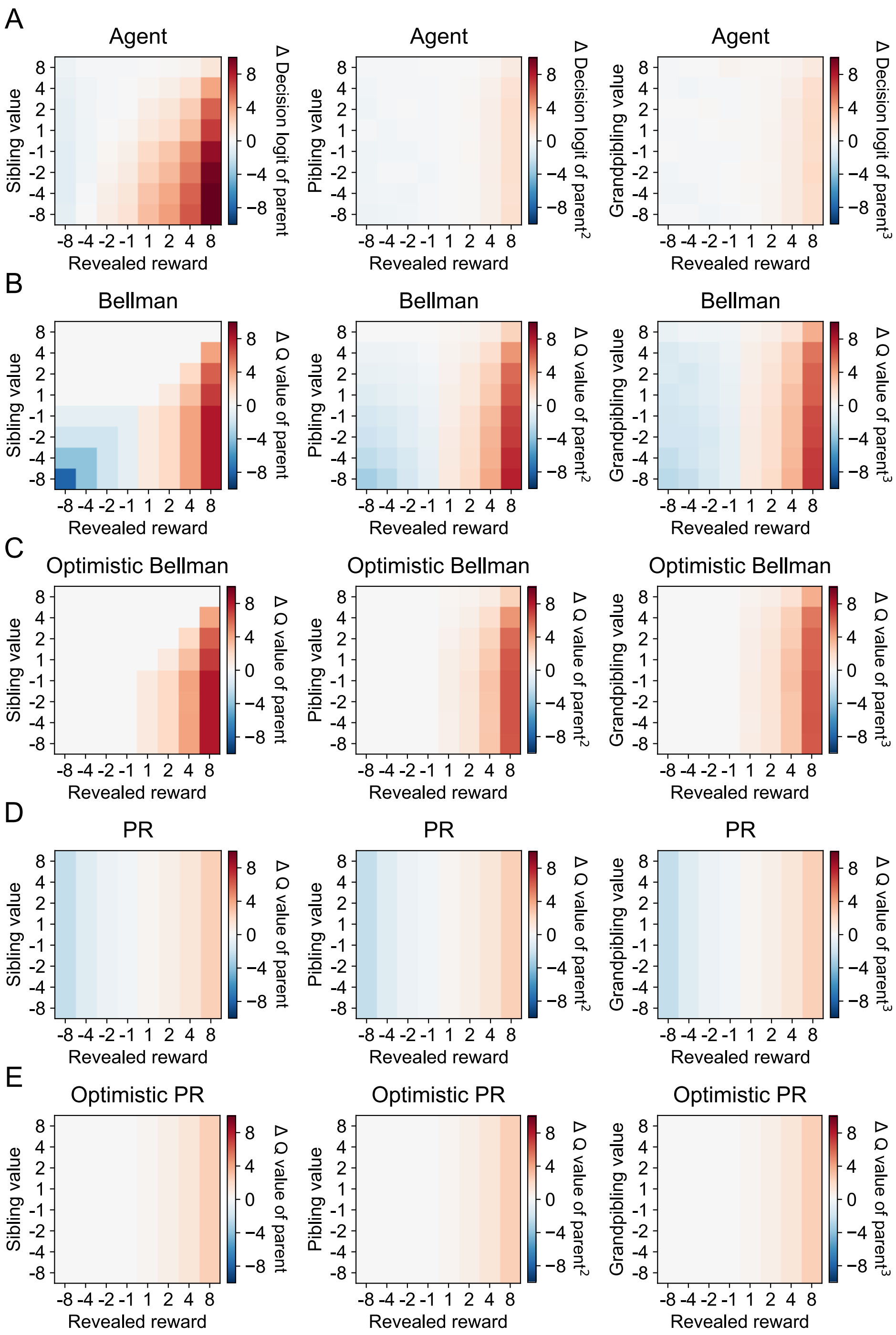

Figure S4: **Comparison of backup strategies with baselines.** We compared the agent's backup strategies against four baselines: (i) Bellman backup: Q values are propagated to the parent by taking the maximum over children; (ii) optimistic Bellman backup: same as (i), but only positive Q values are propagated; (iii): predecessor representation: the agent tracks the identities of all state along the path to the current state, and each query increases of decreases the logits of all the nodes on that path in proportion to the reward at the queried state; and (iv) optimistic predecessor representation: same as (iii), but only positive rewards are propagated. For each scheme, we evaluated how the decision logit of the parent, grandparent, and great-grandparent changes as a function of the revealed reward and the value of the sibling, pibling (i.e., the parent's sibling), and grandpibling (i.e., the grandparent's sibling), respectively. We found that local backups are consistent with optimistic Bellman backups: the parent's logit increases only when the revealed reward exceeds the sibling's value. Non-local backups, however, appear less sensitive to the pibling's or grandpibling's value. They are more consistent with optimistic predecessor representation, in which propagation does not involve taking a maximum over children. We attribute this to memory constraints that limit the agent's ability to perform such maximization over distal relatives.

bioRxiv preprint doi: https://doi.org/10.64898/2026.04.14.718499; this version posted April 16, 2026. The copyright holder for this preprint (which was not certified by peer review) is the author/funder, who has granted bioRxiv a license to display the preprint in perpetuity. It is made available under aCC-BY 4.0 International license.

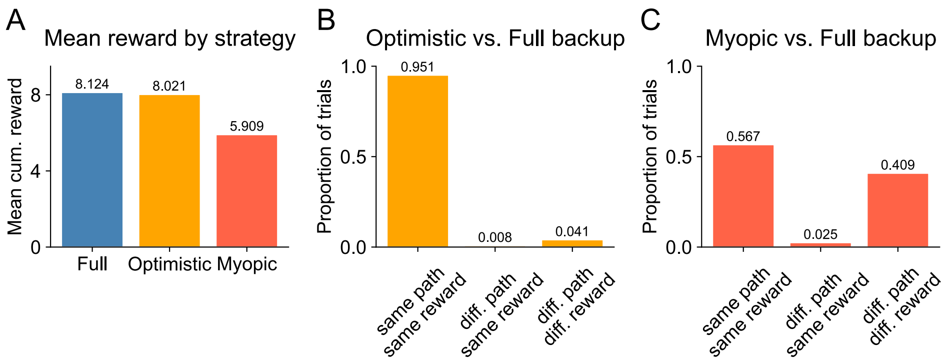

Figure S5: **Optimistic backup is an effective heuristic.** We evaluated whether optimistic backups achieve performance comparable to full Bellman backup. We compared three backup strategies: full Bellman backup, optimistic Bellman backup, and a myopic baseline (no backup), and found that optimistic backup closely matches full backup performance (A), with the two strategies predicting the same chosen path in 95% of trials (B). This confirms that optimistic backup is an effective heuristic and helps explain why the agent's backup strategy shows an optimistic bias.
